# Health system characteristics and COVID-19 performance in high-income countries

**DOI:** 10.1186/s12913-023-09206-z

**Published:** 2023-03-13

**Authors:** Iris Moolla, Heikki Hiilamo

**Affiliations:** Department of Social Research, University of Helsinki, Helsinki, Finland

**Keywords:** Healthcare performance, COVID-19 pandemic, Health system structures, Healthcare financing, Healthcare provision and organization, Health outcomes

## Abstract

**Background:**

The COVID-19 pandemic has shaken everyday life causing morbidity and mortality across the globe. While each country has been hit by the pandemic, individual countries have had different infection and health trajectories. Of all welfare state institutions, healthcare has faced the most immense pressure due to the pandemic and hence, we take a comparative perspective to study COVID-19 related health system performance. We study the way in which health system characteristics were associated with COVID-19 excess mortality and case fatality rates before Omicron variant.

**Methods:**

This study analyses the health system performance during the pandemic in 43 OECD countries and selected non-member economies through three healthcare systems dimensions: (1) healthcare finance, (2) healthcare provision, (3) healthcare performance and health outcomes. Health system characteristics-related data is collected from the Global Health Observatory data repository, the COVID-19 related health outcome indicators from the Our World in Data statistics database, and the country characteristics from the World Bank Open Data and the OECD statistics databases.

**Results:**

We find that the COVID-19 excess mortality and case fatality rates were systematically associated with healthcare system financing and organizational structures, as well as performance regarding other health outcomes besides COVID-19 health outcomes.

**Conclusion:**

Investments in public health systems in terms of overall financing, health workforce and facilities are instrumental in reducing COVID-19 related mortality. Countries aiming at improving their pandemic preparedness may develop health systems by strengthening their public health systems.

## Background

In March 2020, World Health Organization (WHO) declared the coronavirus disease (COVID-19) as a global pandemic. Before that, the first cases were identified in the city of Wuhan in China, and quickly, this novel severe acute respiratory syndrome coronavirus 2 (SARSCoV-2) evolved internationally into a public health emergency [1]. By the end of May 2022, the pandemic had resulted in 523 million confirmed cases and over six million deaths worldwide [2]. The caseloads, case fatality and excess mortality have varied largely across different countries and continents; most of the cases have been reported in high-income countries, such as the OECD countries, in Europe and Americas [2–4], even though these countries have sufficient resources and capacity to invest in their health systems compared to middle- and low-income countries.

Numerous previous studies show that the risk factors for COVID-19 outcomes relate to socioeconomic, environmental, individual demographic and health factors  (e.g. [ 3, 5, 6, 7, 8]). However, the institutional characteristics of health systems have received less attention in connection with the COVID-19 outcomes, and previous findings on the relationship between COVID-19 outcomes and healthcare financing and provision are inconclusive [9–12]. As different variants of coronavirus spread, confirmed number of COVID-19 cases periodically reaches a peak. The relationship between excess deaths and healthcare resources is intuitively related to whether there are enough healthcare resources in a country when the number of diagnoses is very high. The importance of this study emerges from investigating healthcare system structures as a way of understanding differences in COVID-19 outcomes in high-income countries. Thus, the findings may guide policymakers in preparing their national health systems for future pandemics and other health emergencies.

Using publicly available data, this study aims at investigating how the institutional structures of health systems are connected to public health outcomes in terms of COVID-19 excess mortality and case fatality rate in the OECD countries and its non-member economies as it is plausible to compare their ability to provide for care from the resource perspective. We apply healthcare financing characteristics and healthcare system types defined by Moolla et al. [13] to explain the variation in COVID-19 excess mortality and case fatality rates across 43 countries. Thus, this study takes part in the debate on different healthcare systems and their institutional structures. We understand the COVID-19 pandemic as an external shock of health systems and by studying how the institutional structures of health systems link to COVID-19 health outcomes, we can compare how well different health systems have been able to respond to this public health challenge. In this study, we examine the role of healthcare system characteristics in explaining COVID-19 outcomes and thus, we do not aim to provide an overall explanation for varied COVID-19 impacts in different countries.

Healthcare is one determinant of population health and one of the intrinsic goals for healthcare systems is to promote and maintain population health [14]. However, based on the earlier literature, the impact of different health system characteristics to population health outcomes remains unclear. Previous studies have found evidence for the impact of health financing on health outcomes. Higher total healthcare expenditure has been connected to lower mortality and years of life lost, suggesting that increased health spending produces improved health outcomes [15]. Furthermore, findings that higher health expenditure reduces infant mortality supports the evidence that total health expenditure is an important factor in determining health outcomes [16, 17]. Also, in Italy, overall mortality rate increased when the Italian public health system experienced healthcare funding constraints [18].

There are fewer studies examining the connection between health outcomes and the mode of health expenditure. Asiskovitch [19] showed that increased life expectancy at the age of 65 associated with the public mode of healthcare financing. Evidence also suggests that underfunded public health infrastructure created barriers to manage the spread of COVID-19 in the US when the unequal supply of resources left some parts of the US ill-equipped to tackle the epidemic [9], even though the US total healthcare expenditure is the highest in the world [20]. However, higher public health expenditure has been associated with poorer health outcomes [17], and COVID-19 outcomes [10]. More studies on the relationship between the mode of healthcare financing and health outcomes would be needed.

When looking at healthcare provision, earlier studies have found strong associations between population health outcomes and healthcare workforce. Studies have found that greater number of physicians reduces infant mortality [17], and that the higher density levels of doctors, nurses, midwives, and pharmacists improve life expectancy and reduce infant and under-five mortality rates [21]. Several other studies have also found that higher density of healthcare workforce reduces maternal, infant, and under-five mortality rates [22–24]. Furthermore, greater numbers of nurses, midwives and hospital beds have been associated with lower number of COVID-19 cases and mortality [10], and greater number of physicians have been related to lower COVID-19 case fatality rates [10, 11]. However, unexpected findings suggest that better access to essential healthcare services and greater number of physicians are related to negative COVID-19 outcomes [10].

COVID-19 outcomes are found to be related to country clusters with similar economic strength or health status which suggests that country clusters could potentially indicate regions exhibiting similar vulnerabilities to diseases like COVID-19 [10]. One study investigating the relationship between healthcare system types and COVID-19 outcomes found that health system type (social or national health insurance system) is insufficient to explain differences in COVID-19 infection, case fatality and mortality rates [12].

Overall, healthcare system types have not been widely used to the explain differences in health outcomes, but there are a few studies examining the relationship between health outcomes and welfare state regimes —Liberal, Conservative, and Social Democratic — defined by Esping-Andersen [25]. Studies show that health differences by regime are sometimes inconsistent with the welfare regime theory [26]. However, Navarro et al. [27] found that population health was improved by the implementation of policies aimed at reducing social inequalities, which, they said, would explain why health indicators, such as infant mortality, are better in countries governed by pro-distributive political parties. Also, Bambra [28] showed that health status varies across welfare states and infant mortality rates differ significantly between the welfare state regimes connecting higher labour market decommodification with lower infant mortality rates. In addition, social democratic regimes tend to produce best absolute health outcomes and consistently reduce relative health inequalities [29].

Based on the findings of earlier research, we hypothesize that (1) higher total healthcare expenditure is associated with reduced COVID-19 excess mortality and case fatality rates; (2) healthcare system financing types with higher total and public health expenditure have better COVID-19 outcomes; (3) healthcare system provision types with better access to care and higher level of provisional resources are connected with better COVID-19 outcomes; and (4) healthcare system combined types (healthcare financing, provision and outcome dimensions) with high level of resources are associated with reduced COVID-19 excess mortality and case fatality rates. To examine these expectations, next, we move onto describing the research data and methods.

## Methods

### Data

In this study, we analyse the health system performance during the COVID-19 pandemic in 43 high-income countries (high-income and upper-middle-income economies by World Bank lending categories) through their healthcare financing, provision and public health characteristics. The countries were chosen because they represent distinctive healthcare systems from various geographical areas and are OECD’s member states or their selected non-member economies (Brazil, China, Costa Rica, Indonesia, Russia and South Africa) which are OECD’s key collaboration partners and some of the world’s largest economies. Of the non-member economies, India was excluded because of data availability issues. Moreover, due to the data availability and comparability, we restricted our analyses to countries whose healthcare characteristics-related data is collected and made available by the WHO and OECD. Table 1 shows descriptive statistics of the variables used in the analyses.

**Table 1:** Descriptive statistics of the dependent, explanatory and control variables

	(valid obs.)	Mean	SD	Min	Max
*Dependent variables*					
Excess mortality rate (%, until Sep 2021), standardized	40	0	1	-1.4	3.2
Case fatality rate (%, in Sep 2021), standardized	43	0	1	-1.2	4.5
*Health system financing measures*					
Current health expenditure per capita (CHE), /100, standardized	43	0	1	-1.5	3.1
Domestic general government health expenditure as % of CHE, standardized	43	0	1	-2.9	1.3
Out-of-pocket expenditure % of CHE, standardized	43	0	1	-1.4	2.2
*Healthcare system types*					
Financing types	41				
Type 2	19	46.3%			
Type 3	11	26.8%			
Type 4	11	26.8%			
Provision types	41				
Type 1	5	12.2%			
Type 2	7	17.1%			
Type 4	17	41.5%			
Type 5	12	29.3%			
Combined types	41				
Type 1	20	48.8%			
Type 3	11	26.8%			
Type 4	10	24.4%			
*Control variables*					
Population: 80 years old and over % of total population, standardized	43	0	1	-1.9	2.7
DALYs (Disability-Adjusted Life Years) - All causes, both sexes, age-standardized, DALYs per 100 000, standardized	43	0	1	-1.1	4.8
GDP per capita, PPP (current international $), standardized	43	0	1	-1.5	3.6

Sources: [2, 30, 32–36], notes: healthcare system financing type 1, provision type 3 and combined type 2 omitted from the analyses due to small number of countries in those types (only 1 or 2 countries per type)

The COVID-19 related health outcome indicators, COVID-19 excess mortality and case fatality rate, and the indicator of the burden of disease are collected from the Our World in Data statistics database [2, 30]. The database collects data on global problems and includes statistics on the coronavirus pandemic, such as COVID-19 outcomes and vaccinations. The data for the excess mortality is sourced from the Human Mortality Database (HMD) [31] and the World Mortality Dataset (WMD) [32], and for the case fatality from the COVID-19 Data Repository [33]. The HMD and the WMB data are sourced from Eurostat and national statistical agencies (see full list of sources: [31, 32]).

The country characteristics-related data are from the World Bank Open Data database (gross domestic product, GDP) that contains free and open access information on global development [34] and from the OECD statistics database (population share of 80-year-olds and over) that collects statistics on its member and selected non-member states [35]. Measures of healthcare financing are collected from the Global Health Observatory’s (GHO) data repository which includes health-related statistics on the WHO’s member states [36]. We aimed to maximize data comparability and collected GHO data from the year 2018 because most observations were available for that year. The data collection for the healthcare system types is explained in detail by Moolla et al. [13] and descriptions of the different types are found in explanatory variables section.

### Statistical analysis

We applied regression analysis to summarise the influence of explanatory variables upon the COVID-19 outcomes (see [37]). We use both linear bivariate and linear multiple regression methods which are reliable in identifying which explanatory variables have an impact on the COVID-19 excess mortality and case fatality rates and how strong the connections are between the COVID-19 outcomes and the health system characteristics and healthcare system types.

In preparatory analysis we applied one-way analysis of variance (ANOVA) to evaluate whether there is evidence that the group means of countries of different healthcare system types differ in relation to COVID-19 outcomes. We used the Tukey multiple comparison test to investigate which of the means were different when ANOVA test showed evidence for differing group means [38]. In preliminary checks, separate ANOVA tests and regression analyses were applied to excess mortality and case fatality rates that had four different cut off points from the beginning of the COVID-19 pandemic (April 2020, September 2020, March 2021 and September 2021) as we wanted to explore the impact of the pandemic’s different phases on the results. The final models and the presented results use the cumulative COVID-19 excess mortality rate from January 2020 until September 2021 and the COVID-19 case fatality rate at a fixed time point of September 2021 as dependent variables. We considered several regression model specifications with different explanatory variables for both dependent variables. The models we considered to be the most helpful for illustrating associations with COVID-19 outcomes, from which we present results, include models with health system financing characteristics together with healthcare system financing, provision and combined types, defined by Moolla et al. [13]. We decided to exclude the healthcare system outcome types from further analysis as the outcome type 2 included nearly all countries and the COVID-19 outcomes varied largely within that type (see [13]).

### Dependent variables

**Fig. 1:**
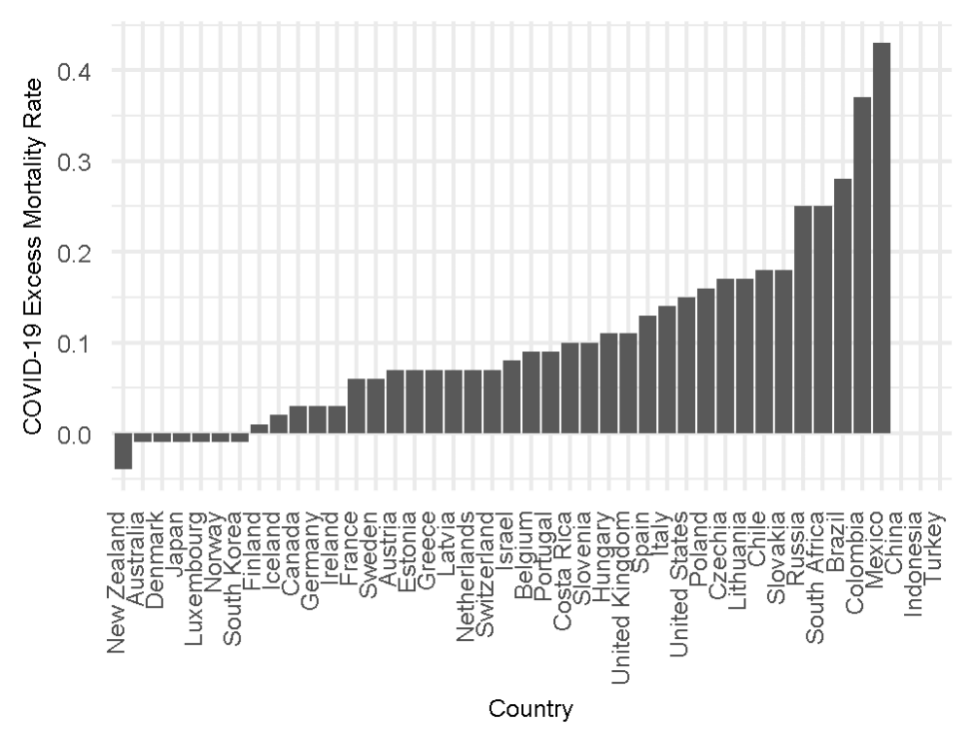
COVID-19 Excess mortality rate by country from Jan 2020 until Sep 2021

**Fig. 2:**
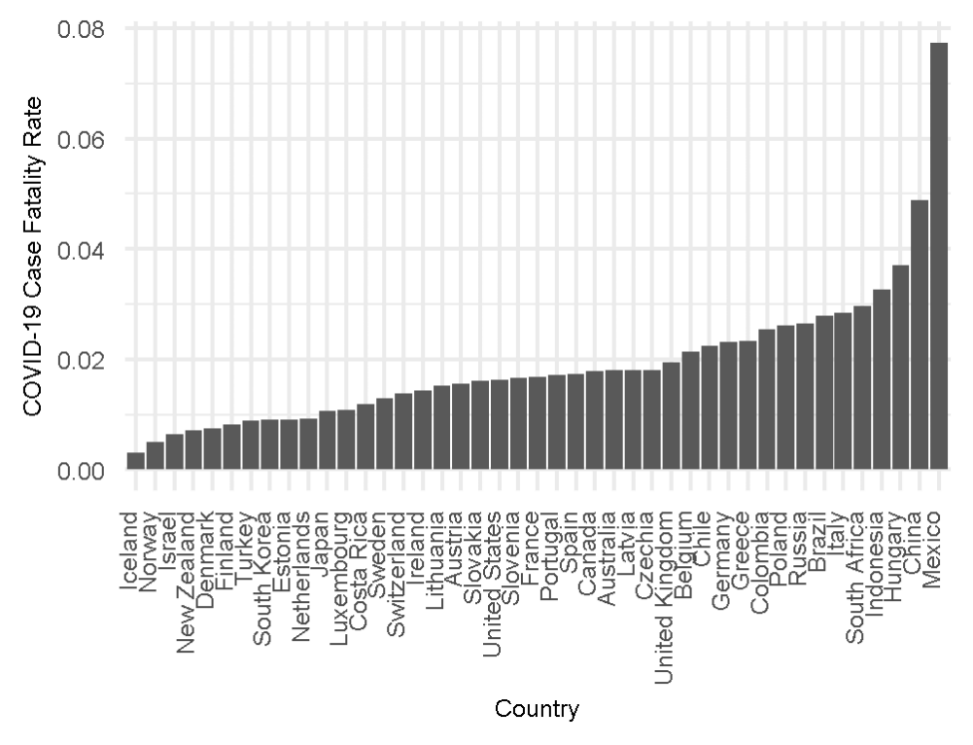
COVID-19 Case fatality rate by country in September 2021

To analyse the impact of health system structures on COVID-19 outcomes, we use COVID-19 excess mortality rate as the main dependent variable and COVID-19 case fatality rate as the second dependent variable for sensitivity checks. Figure 1 shows the COVID-19 excess mortality rate by country from the beginning of 2020 to September 2021 and excludes China, Indonesia and Turkey as the Our World in Data database does not include the excess mortality rate for these countries. Figure 2 presents the COVID-19 case fatality rate by country in September 2021. We look at the COVID-19 outcomes until September 2021 as we wanted our results to be unimpacted by the Omicron variant of COVID-19 detected in November 2021 [39].

The measure of excess mortality provides an estimate of the additional number of deaths during COVID-19 pandemic compared to the deaths if the pandemic had not occurred [2]. The excess mortality rates we use are reported as P-scores which is a more comparable measure of excess mortality as it measures excess mortality as the percentage of the difference between the reported and projected number of deaths divided by the projected number of deaths (see [2]). The baseline for the projected number of deaths is calculated based on the historical deaths data from 2015 to 2019. The P-scores are calculated from the reported deaths data provided for 2020–2022 by HMD [31] and WMD, and from the projected deaths for 2020–2022 provided by WMD [32].

Beaney et al. [40] note excess deaths can be viewed as those caused both directly and indirectly by COVID-19 under the assumption that the incidence of other diseases remains steady over time. This measure is recommended by several international organizations, including the WHO [41] and the European Centre for Disease Prevention and Control [42] as a reliable metric for comparing countries’ COVID-19 outcomes. Previous studies, (e.g. [12, 32, 43–45]) have used the measure of excess mortality to compare COVID-19 outcomes cross-nationally.

The all-cause excess deaths measure has several advantages which Corrao et al. [43] describe as follows: all-cause excess mortality (1) is not based on clinical diagnosis of the cause of death and thus, it is unaffected by incompatibility of different diagnostic criteria; (2) is unaffected by between-country differences in age and comorbidity structure as each country’s values are compared with itself; and (3) is a more accurate measure than the COVID-19-specific mortality rate as there is less random uncertainty connected to its monitoring and demographic distribution. In addition, it overcomes the variation between countries in misclassification of the cause of death in death certificates, in reporting and testing for COVID-19. Thus, it presents comprehensively the full impact of the pandemic in populations health status [12]. However, its limitations lie in explaining differences between countries as it does not differentiate the cause-specific mortality. Where data collection and reporting systems are timely and comprehensive, excess mortality gives a summary measure of the impact of different pandemic responses [40].

The COVID-19 case fatality rate instead, is measured as the number of confirmed COVID-19 deaths divided by the number of confirmed COVID-19 cases. Rather than being constant, the case fatality rate reflects the severity of the disease in a particular context, at a particular time and in a particular population [2]. Case fatality rate’s limitations connect to the overestimation and underestimation of the risk of death; the number of undiagnosed people will cause the rate to overestimate and living diagnosed people will cause the rate to underestimate the true risk of death [2], and that it depends on the accurate determination of causes of death [12]. However, it can be used as a measure of disease severity and its comparatively high rates indicate relatively poor COVID-19 outcomes. This measure is also widely used in studies on COVID-19 outcomes (e.g. [4, 10–12]).

### Explanatory variables

For explaining the COVID-19 outcomes, we use measures of health system characteristics and healthcare system types. Previous research has studied healthcare financing [15–19], healthcare provision [21–24] and health system categorisations [12, 28] in relation to health outcomes and thus, we can expect the structural differences of national health systems relate to the COVID-19 outcomes. First, we apply three indicators to represent the financing characteristics of health systems. The measure of current health expenditure (CHE) per capita indicates the level of resources invested in health systems [46]. We measure it in $US per capita by using purchasing power parity and its values are divided by 100 to scale them similarly as the other financing measures. We use the indicator of general government health expenditure as a percentage of CHE to measure public health expenditure. It reflects the government’s role in controlling healthcare payments and the extent of public responsibility in organizing healthcare [47]. The indicator of households’ out-of-pocket payments as a percentage of CHE represents the healthcare costs and financial burden of private households as well as the role of the markets in healthcare [46, 47].

Second, we use the healthcare system financing, provision and combined types to explain the variation in COVID-19 outcomes between countries. Tables 2, 3 and 4 describe each of the healthcare system type whose more specified details and formation with cluster analysis are explained by Moolla et al. [13] and we follow their naming of the system types. The types are used as a tool to examine the relationship between COVID-19 outcomes and the health system structures and we acknowledge that other ways of grouping healthcare systems could be used in this context as well. However, these types are useful estimates of different healthcare system types which enable investigating different dimensions of health systems (financing, provision and health outcomes) separately and combined.

**Table 2:** Description of the healthcare system financing types

Types	Current health expenditure^a^	Public health expenditure^b^	Private out-of-pocket health expenditure^c^	Countries
Type 1	The highest level	The lowest level	Average level	Switzerland, the US
Type 2	High level	The highest level	The lowest level	Australia, Austria, Belgium, Canada, Czechia, Denmark, Finland, France, Germany, Iceland, Ireland, Japan, Luxembourg, the Netherlands, New Zealand, Norway, Slovenia, Sweden, the UK
Type 3	The lowest level	Low level	The highest level	Brazil, Chile, China, Greece, Indonesia, Latvia, Lithuania, Mexico, Portugal, South Korea, Russia
Type 4	Low level	Average level	Average level	Colombia, Costa Rica, Estonia, Hungary, Israel, Italy, Poland, Slovakia, South Africa, Spain, Turkey

Source: [13]. Notes: a $US per head of population by using purchasing power parity; b general government health expenditure as a percentage of CHE; c household’s out-of-pocket payments as a percentage of CHE

Healthcare system financing types categorize countries based on their current total, public and private out-of-pocket health expenditure characteristics. Higher CHE reflects better financial capacity of healthcare systems to provide health services; higher share of public health expenditure reflects larger public responsibility over healthcare financing whereas higher share of private expenditure presents larger role of private households in covering healthcare costs [47]. For example, the financing type 1 represents health systems of OECD countries and their member economies that have the highest level of CHE, the lowest level of government financing and an average share of private out-of-pocket payments (see Table 2).

**Table 3:** Description of the healthcare system provision types

Types	Primary care delivery and gatekeeping	Number of doctors^a^ and hospital beds^b^	Coverage of essential services	Countries
Type 1	Primary care mainly private, non-existent gatekeeping	Average number of doctors, the highest number of hospital beds	The highest level	Austria, Czechia, Japan, Luxembourg, South Korea
Type 2	Primary care public-private mix, non-existent gatekeeping	Average number of doctors, low number of hospital beds	Average level	China, Greece, Iceland, Russia, Sweden, Turkey, the US
Type 3	Primary care public-private mix, moderate gatekeeping	The lowest level	The lowest level	Indonesia, South Africa
Type 4	Primary care mainly private, strong to moderate gatekeeping	Average level	High level	Australia, Belgium, Canada, Denmark, Estonia, France, Germany, Hungary, Ireland, Latvia, the Netherlands, New Zealand, Norway, Poland, Slovakia, Switzerland, the UK
Type 5	Primary care mainly public or public-private mix, strong gatekeeping	The highest number of doctors, low number of hospital beds	Average level	Brazil, Chile, Colombia, Costa Rica, Finland, Israel, Italy, Lithuania, Mexico, Portugal, Slovenia, Spain

Source: [13]. Notes: a total number of hospital beds per 10,000 population; b density of practising doctors per 10,000 population

Table 3 presents the healthcare system provision types which are formed based on the availability and supply of health services different health systems. Primary care delivery reflects the public-private-mix in organizing primary healthcare supply [48]; gatekeeping reflects how specialist care access is controlled [49]; the number of doctors and hospital beds indicate health service availability and accessibility [49]; and the coverage of essential health services indicates the level of resources and accessibility of essential care, such as maternal and child healthcare [36]. For example, provision type 1 represents health systems whose primary health services are mainly organized in the private sector, gatekeeping is non-existent, the number of doctors is average, the number of hospital beds and the coverage of essential health services are high.

**Table 4:** Description of the healthcare system combined types

Types	Financing	Provision	Health outcomes	Countries
Type 1	The highest level of CHE; the highest level of public health expenditure; the lowest level of private health expenditure	Primary care mainly private; strong to low gatekeeping; high number of hospital beds and doctors; high coverage of essential services	The best population health outcomes	Australia, Austria, Belgium, Canada, Czechia, Denmark, France, Germany, Iceland, Ireland, Japan, Luxembourg, Netherlands, New Zealand, Norway, South Korea, Sweden, Switzerland, the UK, the US
Type 2	The lowest level of CHE; the lowest level of public health expenditure; low level of private health expenditure	Primary care public-private mix; moderate gatekeeping; low number of hospital beds and doctors; low coverage of essential services	The worst population health outcomes in terms of child and maternal health	Indonesia, South Africa
Type 3	Low level of CHE; low level of public health expenditure; high level of private health expenditure	Primary care mainly public or public-private mix; moderate to low gatekeeping; low number of hospital beds; high number of doctors; average to low coverage of essential services	Population health outcomes good expect high maternal mortality	Brazil, Chile, Colombia, Costa Rica, Finland, Israel, Italy, Mexico, Portugal, Spain
Type 4	Low level of CHE; average level of public health expenditure; high level of private health expenditure	Primary care mainly private or public-private mix; strong to low gatekeeping; high number of hospital beds; average number of doctors; low coverage of essential services	Population health outcomes average, cancer mortality and differences in life expectancy between men and women the highest	China, Greece, Estonia, Hungary, Latvia, Lithuania, Poland, Slovakia, Slovenia, Russia, Turkey

Source: [13]

The healthcare system combined types reflect health systems’ financing, provision and population health outcomes characteristics. The health financing and provision characteristics are the same as described above whereas population health outcomes are measured as measles-vaccine coverage among 1-years-olds, life expectancy, distribution of life expectancy, maternal and cancer mortality. For example, the first combined type represents health systems which are characterized by the lowest share of out-of-pocket expenditure, the highest level of CHE and government health expenditure. The healthcare supply is on a high level. Primary care is mainly organized in the private sector, but gatekeeping practices vary from strict to non-existent. These countries produce the best population health outcomes in terms of high measles-vaccine coverage, high life expectancy, small differences in life expectancy between males and females, low maternal and cancer mortality. The other three healthcare system combined types and the countries representing them are presented in Table 4.

### Control variables

The country characteristics-related data, GDP per capita, DALYs and population share of 80-year-olds and over are used as control variables in the regression models (see Table 1 for descriptive statistics). The data for GDP per capita and population share of 80-year-olds and over is from the year 2020 and for DALYs from the year 2019.

COVID-19 has disproportionally affected older and vulnerable populations [45], and thus, we can expect varying age and burden of disease patterns of COVID-19 outcomes based on a population’s demographic and epidemiological profile. Advanced population age has shown to have a positive relationship with the number of COVID-19 cases and deaths [10, 50]. Instead of population share aged 65 or over, we use the share of population aged 80 or over as a control variable as the age group of 65-year-olds and over might be too broad to disentangle the impact of old age on COVID-19 outcomes.

Together with advanced age, underlying health conditions have been associated with higher risk for severe COVID-19 infection [51]. Thus, we control for the burden of disease by using the measure of disability-adjusted life years which assesses the burden of disease or health condition borne by individuals in different populations [30]. In addition, we control for GDP per capita as it has been connected with COVID-19 health outcomes [50], and it reflects the wealth of the countries and thus, the possibilities to invest in their healthcare sector.

In preliminary checks, we tested the measures of the population share aged 65 or over, the population share fully vaccinated against COVID-19, Gini index, income share held by lowest 20% in a population, financial austerity of health systems (changes in countries’ shares of out-of-pocket and private health expenditure within the past five years), the number of hospital beds and doctors. In final models these measures we left out as they did not present a statistically significant relationship with the outcome variables or improve the model fits.

## Results

In Tables 5 and 6, we present the regression results of COVID-19 outcomes explained by the health system financing characteristics. Table 5 shows the results for the models with excess mortality rate and Table 6 with case fatality rate as the dependent variable. First, the tables present models only with the financing measures that we then control for with measures of DALYs per 100 000, GDP per capita and population share of 80-year-olds and over. In addition, for models with excess mortality rate, Mexico, South Africa and Colombia are controlled as they were outliers and high-leverage points for the regression models due to their high excess mortality rates and low share of population aged 80 or over. In addition, South Africa and Mexico show high numbers of DALYs. For the models with case fatality rate, Mexico and China are controlled due to their high case fatality rates.

**Table 5:** Regression results with health system financing characteristics: Excess mortality rate

	Model 1a	Model 1b	Model 1c	Model 2a	Model 2b	Model 2c	Model 3a	Model 3b	Model 3c
	Β (sd)	Β (sd)	Β (sd)	Β (sd)	Β (sd)	Β (sd)	Β (sd)	Β (sd)	Β (sd)
(Intercept)	0.00	0.00	-0.05	0.00	0.00	-0.04	0.00	0.00	-0.05
	(0.13)	(0.12)	(0.09)	(0.15)	(0.11)	(0.09)	(0.14)	(0.11)	(0.08)
Current health expenditure	-0.55***	-0.34*	-0.15						
	(0.14)	(0.13)	(0.10)						
Out-of-pocket expenditure				0.37*	0.17	-0.00			
				(0.15)	(0.12)	(0.11)			
Government health expenditure							-0.45**	-0.16	-0.11
							(0.14)	(0.12)	(0.09)
Population 80 years or over		-0.22	0.00		-0.28*	-0.04		-0.25	-0.03
		(0.14)	(0.10)		(0.13)	(0.10)		(0.13)	(0.10)
DALY		0.33*	0.77***		0.26	0.69***		0.20	0.64***
		(0.15)	(0.17)		(0.14)	(0.18)		(0.15)	(0.17)
GDP					-0.36*	-0.23*		-0.42**	-0.21*
					(0.14)	(0.11)		(0.13)	(0.10)
Mexico			2.15***			2.08**			2.02***
			(0.58)			(0.58)			(0.55)
South Africa			-2.53*			-2.38*			-2.19*
			(0.93)			(1.04)			(0.90)
Colombia			2.23***			2.04**			2.13***
			(0.57)			(0.58)			(0.55)
R2	0.31	0.50	0.77	0.14	0.58	0.79	0.20	0.58	0.80
adj. R2	0.29	0.45	0.73	0.11	0.53	0.75	0.18	0.53	0.76
logLik	-48.91	-42.57	-26.73	-53.30	-38.96	-24.94	-51.76	-39.12	-23.99
AIC	103.83	95.14	69.46	112.59	89.92	67.87	109.51	90.23	66.00
BIC	108.90	103.58	83.00	117.66	100.05	83.07	114.56	100.37	81.19

Notes: ***p < 0,001, ** p < 0,01, *p < 0,05; (standard deviation), N = 40, GDP not a control variable for models 1b and 1c due to high correlation with current health expenditure measure

**Table 6:** Regression results with health system financing characteristics: Case fatality rate

	Model 4a	Model 4b	Model 4c	Model 5a	Model 5b	Model 5c	Model 6a	Model 6b	Model 6c
	Β (sd)	Β (sd)	Β (sd)	Β (sd)	Β (sd)	Β (sd)	Β (sd)	Β (sd)	Β (sd)
(Intercept)	0.00	0.00	-0.16	0.00	0.00	-0.15	0.00	0.00	-0.15
	(0.14)	(0.14)	(0.08)	(0.13)	(0.12)	(0.08)	(0.14)	(0.13)	(0.08)
Current health expenditure	-0.45**	-0.31	-0.16						
	(0.14)	(0.16)	(0.09)						
Out-of-pocket expenditure				0.52***	0.40**	0.11			
				(0.13)	(0.14)	(0.10)			
Government health expenditure							-0.45**	-0.26	-0.12
							(0.14)	(0.15)	(0.09)
Population 80 years or over		-0.04	0.17		-0.09	0.13		-0.06	0.15
		(0.17)	(0.10)		(0.15)	(0.10)		(0.16)	(0.10)
DALY		0.27	0.35**		0.25	0.32***		0.14	0.28*
		(0.17)	(0.10)		(0.16)	(0.10)		(0.18)	(0.11)
GDP					-0.12	-0.12		-0.29	-0.16
					(0.17)	(0.10)		(0.16)	(0.09)
Mexico			4.38***			4.14***			4.25***
			(0.53)			(0.56)			(0.53)
China			2.49***			2.30***			2.35***
			(0.54)			(0.55)			(0.54)
R2	0.21	0.27	0.78	0.27	0.41	0.79	0.20	0.34	0.79
adj. R2	0.19	0.22	0.75	0.25	0.33	0.75	0.18	0.27	0.75
logLik	-55.55	-53.71	-28.39	-53.74	-49.27	-27.25	-55.63	-51.61	-26.99
AIC	117.11	117.41	70.78	113.49	110.54	70.50	117.25	115.22	69.97
BIC	122.39	126.22	83.11	118.77	121.11	84.59	122.54	125.78	84.06

Notes: ***p < 0,001, ** p < 0,01, *p < 0,05; (standard deviation), N = 43, GDP not a control variable for models 1b and 1c due to high correlation with current health expenditure measure

We see statistically significant relationships between all the financing characteristics and excess mortality and case fatality rate. The connections have the same directions with both dependent variables with and without control variables (except Model 2c). We find that higher CHE and public health expenditure are associated with lower excess mortality and case fatality rates, whereas a higher share of out-of-pocket expenditure is related to higher excess mortality and case fatality rates. The financing characteristics seem to explain around 15 to 30% of the variation in excess mortality and case fatality rates. However, when the controls are added, the significant connections mostly decrease or disappear.

After controlling for other variables and the outlier countries, the results indicate that the population share of 80-year-olds and over has no statistically significant relationship with COVID-19 outcomes. However, in Models 1c-6c the negative relationships between advanced age and COVID-19 outcomes diminishes or becomes positive when the country-controls are added since they represent countries with younger populations but with high excess mortality and case fatality rates. DALYs have a strong and positive relationship with excess mortality and case fatality, indicating that the greater previous burden of disease is associated with higher numbers of COVID-19 excess mortality and case fatality. GDP per capita, net-of-controls, has a strong and negative relationship with excess mortality and also, a negative relationship with case fatality: higher GDP is associated with lower excess mortality and case fatality rates.

Tables 7 and 8 present the results of linear regression analyses in which excess mortality rate and case fatality rate are explained by the healthcare system financing, provision and combined types. Financing type 1, provision type 3 and combined type 2 were excluded from the models since they included only two countries and can be seen as outlier countries. For models with excess mortality rate, Mexico and Colombia are controlled as they were outliers and high-leverage points for the regression models due to their high excess mortality rates, whereas for the models with case fatality rate, Mexico and China are controlled due to their high case fatality rates.

Compared to the second financing type, financing types 3 and 4 have higher excess mortality and case fatality rates. It appears that health systems with a lower share of private health expenditure and a high level of CHE and public health expenditure produce better COVID-19 outcomes (such as systems in the financing type 2) than health systems with a higher share of private out-of-pocket payments, a lower level of CHE and public financing.

**Table 7:** Regression results with healthcare system types: Excess mortality rate

	Model 7a	Model 7b	Model 8a	Model 8b	Model 9a	Model 9b
	Β (sd)	Β (sd)	Β (sd)	Β (sd)	Β (sd)	Β (sd)
(Intercept)	-0.60**	-0.60**	-0.58	-0.58	-0.57**	-0.57***
	(0.19)	(0.15)	(0.39)	(0.30)	(0.18)	(0.14)
Financing type 3	1.25***	0.94**				
	(0.34)	(0.27)				
Financing type 4	1.15**	0.92**				
	(0.33)	(0.26)				
Provision type 2			0.65	0.65		
			(0.55)	(0.43)		
Provision type 4			0.17	0.17		
			(0.44)	(0.34)		
Provision type 5			1.26*	0.83*		
			(0.47)	(0.37)		
Combined type 3					1.26***	0.77**
					(0.31)	(0.25)
Combined type 4					0.89*	0.89**
					(0.34)	(0.26)
Mexico		2.82***		2.91***		2.95***
		(0.68)		(0.71)		(0.64)
Colombia		2.26**		2.33**		2.37***
		(0.67)		(0.71)		(0.64)
N	38	38	39	39	39	39
R2	0.35	0.65	0.28	0.59	0.34	0.66
adj. R2	0.32	0.61	0.21	0.53	0.30	0.62
logLik	-46.01	-34.16	-47.99	-36.93	-46.26	-33.48
AIC	100.02	80.32	106.00	87.85	100.52	78.96
BIC	106.57	90.14	114.31	99.50	107.18	88.94

Notes: ***p < 0,001, ** p < 0,01, *p < 0,05; (standard deviation), Reference categories: Financing type 2, Provision type 1, Combined type 1

When comparing provision types, we notice that provision type 1 is associated with the best COVID-19 outcomes in relation to the other provision types. Healthcare systems of provision type 1 differ from the other provision types especially in terms of greater number of hospital beds and better coverage of essential health services. However, the results with provision types do not indicate strong associations with COVID-19 outcomes.

**Table 8:** Regression results with healthcare system types: Case fatality rate

	Model 10a	Model 10b	Model 11a	Model 11b	Model 12a	Model 12b
	Β (sd)	Β (sd)	Β (sd)	Β (sd)	Β (sd)	Β (sd)
(Intercept)	-0.43*	-0.43**	-0.48	-0.48	-0.43*	-0.43**
	(0.21)	(0.13)	(0.45)	(0.26)	(0.21)	(0.12)
Financing type 3	1.20**	0.61*				
	(0.35)	(0.24)				
Financing type 4	0.48	0.48*				
	(0.35)	(0.22)				
Provision type 2			0.55	0.18		
			(0.59)	(0.36)		
Provision type 4			0.28	0.28		
			(0.51)	(0.30)		
Provision type 5			0.77	0.39		
			(0.53)	(0.32)		
Combined type 3					0.78*	0.36
					(0.36)	(0.21)
Combined type 4					0.73	0.50*
					(0.37)	(0.22)
Mexico		4.33***		4.60***		4.58***
		(0.62)		(0.61)		(0.58)
China		2.13**		2.61***		2.23***
		(0.62)		(0.64)		(0.58)
N	41	41	41	41	41	41
R2	0.24	0.71	0.07	0.70	0.14	0.73
adj. R2	0.2	0.67	-0.01	0.65	0.10	0.70
logLik	-52.97	-33.48	-56.26	-33.20	-54.55	-30.87
AIC	113.94	78.96	122.51	80.40	117.09	73.73
BIC	120.79	89.24	131.08	92.39	123.95	84.02

Notes: ***p < 0,001, ** p < 0,01, *p < 0,05; (standard deviation), Reference categories: Financing type 2, Provision type 1, Combined type 1

Finally, compared to the healthcare system combined type 1, the combined types 3 and 4 seem to have higher rates of excess mortality and case fatality. Health systems with a low share of out-of-pocket expenditure, and a high level of CHE, government health expenditure, healthcare employment, hospital beds and essential health services seem to produce better COVID-19 related health outcomes (systems such as combined type 1). The health systems of the combined type 1 also produce the best other population health outcomes (high measles-vaccine coverage among 1-years-olds, high life expectancy, more equal distribution of life expectancy, low maternal and cancer mortality) which may indicate that health systems that had a good performance even before COVID-19 pandemic, have also been able to perform better during the COVID-19 pandemic.

## Discussion

### Findings on COVID-19 outcomes and health system characteristics

At the beginning of this paper, we hypothesized that (1) higher total healthcare expenditure is associated with reduced COVID-19 excess mortality and case fatality rates; (2) healthcare system financing types with higher total and public health expenditure produce better COVID-19 outcomes; (3) healthcare system provision types with better access to care and higher level of provisional resources are connected with better COVID-19 outcomes; and (4) healthcare system combined types with high level of resources are associated with reduced COVID-19 excess mortality and case fatality rates. Our findings support these expectations and find support from earlier research on the relationship between health outcomes and health system characteristics. The results suggest that higher health financing and provision resources contribute to lower COVID-19 excess mortality and case fatality rates. Also, better public health outcomes prior to COVID-19 pandemic seem to indicate better COVID-19 outcomes. Our findings indicate that strengthening the overall health financing, workforce and facilities of national public health systems reduces COVID-19 related mortality.

Our findings indicate that lower COVID-19 excess mortality and case fatality rates are associated with higher total healthcare expenditure which has also been connected to better health outcomes such as lower mortality rates [15–18]. The relationship between health outcomes and private and public health expenditure is still understudied and the findings inconclusive; however, our results suggest that countries with higher public and lower private health expenditure have produced better COVID-19 outcomes. In addition, we find that better access to care and higher level of provisional resources such as number of hospital beds are connected to lower COVID-19 excess mortality and case fatality rates. Earlier studies have also shown that higher level of health workforce is associated with better population health outcomes [17, 21–24]. The higher level of health workforce and facilities have also been connected to better COVID-19 outcomes [10, 11].

While the relationship between healthcare system types and COVID-19 outcomes, or other health outcomes, is underexplored in previous literature, our findings indicate that healthcare system types provide a useful tool for understanding differences in COVID-19 outcomes in high-income countries. Even though Simoes et al. [12] did not find support for the type of health insurance system in explaining COVID-19 outcomes, other studies have indicated that clustering of countries helps to explain country differences in COVID-19 outcomes, or other health outcomes [10, 28, 29]. However, these studies have connected these differences in health outcomes to countries’ similar vulnerabilities to diseases [10], implementation of policies aimed at reducing social inequalities [27], and promotion of social protection and provision of public goods [28, 29]. Our application of healthcare system types suggests that COVID-19 health outcomes are impacted by the health system structures. We find evidence that health systems with low private health expenditure, high total and government health expenditure, a high level of health workforce and facilities produce better COVID-19 related health outcomes. Also, health systems that had produced good population health outcomes before the pandemic, have also had better performance in terms of COVID-19 excess mortality and case fatality. Even though the healthcare system types contain health systems with varied resources and national characteristics, they show us a compelling way of understanding differences in COVID-19 outcomes from the health system structures perspective. Altogether, our findings indicate that publicly funded health systems with greater level of resources have been able to perform better during the COVID-19 pandemic than systems with greater private health expenditure and lower level of resources.

### Limitations

Limitations of this study relate to the reliability of the data and the complexity of measuring COVID-19 health outcomes. As noted earlier, the databases from which the data for constructing the excess mortality and case fatality rates are sourced, are from several different national and international sources. Thus, the data collection and reporting methods vary across countries. For the excess mortality indicator, the coverage and reliability of the data varies across countries, and some of the countries might have reported incomplete mortality numbers underestimating the excess mortality rates (see [32]). The same caveat applies to the case fatality estimates as well; it is possible that various reporting methods result in incomplete estimates. However, the databases for collecting COVID-19 related information list their original data sources rigorously and explain their data collection and estimate calculation methods in detail. To the best of our knowledge, the data on COVID-19 excess mortality and case fatality has been collected and reported by following reliable research practices.

Due to the complexity of the COVID-19 pandemic and used COVID-19 indicators, COVID-19 outcomes cannot fully be explained with healthcare characteristics. COVID-19 is likely to be connected to geographical and welfare state related factors such as social protection and different reactions to buffer the pandemic; for example, the different trajectories of Sweden and other Nordic countries (strict versus loose COVID-19 response) [52], or situation in countries which followed extreme lock-down policies nationally (e.g. New Zealand) or locally (e.g. China). The excess mortality and case fatality rates are also related to factors outside healthcare systems and depend on larger societal, environmental, economic and political factors [45, 53]. Also, different ways of counting COVID-19 deaths and cases might impact the comparability of the indicators across countries [54], for example, at the beginning of the pandemic, nursing homes’ COVID-19 deaths in the US were not reported as part of the country’s overall COVID-19 deaths [55].

### Limitations on the measure of COVID-19 excess mortality

Previous research has found evidence for alternative drivers of mortality in societies that might have impacted the excess mortality during the pandemic. Studies suggest that diminished healthcare use might have affected the excess mortality rates (e.g.[ 56, 57]), which, however, is difficult to prove and the effect of changes in healthcare use on excess mortality might also be greater after the first years of the pandemic [45]. Researchers have showed increased rates of anxiety and depression during the pandemic, which might have led to increased deaths from suicide [58–60]. Deaths from some chronic conditions such as ischemic heart disease or chronic respiratory disease decreased in 2020 [57], which might have occurred because frail individuals who suffered from these conditions, already died earlier that year from COVID-19 [45]. Still, especially in the Northern Hemisphere, decreases in flu and common respiratory virus deaths (decreases in cases of 80% or more), provide the most compelling evidence of changes in cause-specific mortality during the pandemic [45]. In addition, social distancing and other pandemic restrictions might have decreased deaths from injuries, such as road accidents [61, 62]. COVID-19 excess mortality is affected by these changes in baseline patterns of all-cause mortality making it challenging to differentiate how much excess mortality is due to SARS-CoV-2 infection or other societal, economic, or behavioural changes associated with the pandemic [45].

### Limitations on the measure of COVID-19 case fatality

The COVID-19 case fatality rate instead is affected by the reporting of the number of COVID-19 deaths in healthcare, as listing COVID-19 as the cause of death requires a positive SARS-CoV-2 test which affects the official counts especially when the testing capacity is low [45]. Especially in long-term care facilities, COVID-19 deaths have been underreported among older individuals in high-income countries (e.g.[ 54, 63]). Also, the quality of systems for registering and definitions used for counting COVID-19 deaths varied across countries, as well as different political considerations and social, economic, and behavioural responses to the pandemic, including strict lockdowns, appear to have affected the accurate reporting of COVID-19 deaths [45]. It is necessary to emphasize that our results describe COVID-19 outcomes before the emergence of the Omicron variant which dramatically changed the global pandemic trajectories.

However, we are not trying to provide an overall explanation for COVID-19 outcomes but to disentangle the possible role of healthcare system characteristics in tackling pandemics such as COVID-19. Our analyses consider the impact of societal characteristics in terms of GDP, DALYs and population share over 80-year-olds on COVID-19 outcomes and the results show that these factors are connected to the outcomes. Previous studies also find similar results of advanced population age and underlying health conditions being connected to higher COVID-19 caseloads and overall mortality [10, 50]. However, our results indicate that higher GDP per capita is associated with better COVID-19 outcomes which are inconsistent with Chaudry’s et al.’s findings which according to them might reflect more widespread testing, greater reporting transparency and better national surveillance systems in countries with a higher GDP [50].

## Conclusion

To conclude, our findings suggest that before Omicron variant COVID-19 excess mortality and case fatality rates were systematically associated with healthcare systems’ financing, provision and public health outcome characteristics, and that healthcare system types provide a tool for understanding differences in COVID-19 outcomes in high-income countries. Although the study setting did not allow causal inferences, our results suggest that investments in public health systems in terms of overall financing and a high level of health workforce and facilities are instrumental in reducing COVID-19 related mortality. Countries aiming at improving their pandemic preparedness may develop health systems by strengthening their public health systems.

## Data Availability

All data collected and analyzed during the current study are publicly available in the following repositories: the Our World in Data database [https://ourworldindata.org/excess-mortality-covid]; [https://ourworldindata.org/burden-of-disease], the Human Mortality Database [www.mortality.org]; [www.humanmortality.de], the World Mortality Dataset [10.7554/eLife.69336], the World Bank Open Data database [https://data.worldbank.org/], the OECD Health Statistics database [10.1787/data-00547-en], and the Global Health Expenditure Database (GHO) [https://apps.who.int/nha/database/Home/Index/en].

## List of abbreviations

COVID-19: Coronavirus disease
WHO: World Health Organization
SARSCoV-2: severe acute respiratory syndrome coronavirus 2
OECD: The Organisation for Economic Co-operation and Development
US: the United States
HMD: the Human Mortality Database
WMD: the World Mortality Dataset
GDP: gross domestic product
GHO: the Global Health Observatory
ANOVA: analysis of variance
CHE: current health expenditure
DALY: disability-adjusted life years
